# Mavacamten and Systolic Dysfunction in the FDA Adverse Event Reporting System: A Disproportionality Analysis With an Indication-Restricted Comparator

**DOI:** 10.7759/cureus.117905

**Published:** 2026-10-14

**Authors:** João Marcelo Sá Pinto L De Oliveira, Laura B Lima, Lorenza Sobrinho B Santos, Rafael Castione de Paula S Lima, Humberto G Moreira

**Affiliations:** 1 School of Medicine, Instituto de Educação Médica - UniFacid, Teresina, BRA; 2 School of Medicine, Instituto Master de Ensino Presidente Antônio Carlos (IMEPAC), Araguari, BRA; 3 School of Medicine, Centro Universitário FIPMoc, Montes Claros, BRA; 4 School of Medicine, Faculdade de Ciências da Saúde de Barretos, Barretos, BRA; 5 School of Medicine, Federal University of Goiás, Goiânia, BRA

**Keywords:** cardiac myosin inhibitor, confounding by indication, disproportionality analysis, faers, hypertrophic cardiomyopathy, mavacamten, pharmacovigilance

## Abstract

Introduction: Mavacamten, a cardiac myosin inhibitor for obstructive hypertrophic cardiomyopathy (HCM), is dispensed under a Risk Evaluation and Mitigation Strategy requiring serial echocardiography for reduced ejection fraction. Because HCM itself causes systolic dysfunction and heart failure, a disproportionality estimate drawn from the whole database confounds the drug with its indication. We examined whether the reporting signal persists when the comparator is restricted to the same indication.

Methods: We analysed the US Food and Drug Administration (FDA) Adverse Event Reporting System (FAERS) through the openFDA interface, using the snapshot dated July 30, 2026. The composite outcome comprised 12 Medical Dictionary for Regulatory Activities (MedDRA) Preferred Terms covering systolic dysfunction and heart failure. Disproportionality was measured by reporting odds ratio (ROR), proportional reporting ratio (PRR), and information component (IC) against two pre-specified comparators: the whole database, which yields the crude, indication-unadjusted estimate, and reports carrying an explicit HCM indication, which is the comparison that addresses the study question. The interval from treatment start to report receipt was described with a Weibull model.

Results: Of 5,083 reports naming mavacamten, 379 (7.5%) met the composite outcome. Against the whole database, the signal was strong (ROR 8.29, 95% confidence interval (CI) 7.47-9.21; PRR 7.75; IC025 2.78). Against the 527 reports sharing an HCM indication, it was absent (ROR 0.80, 95% CI 0.58-1.09; PRR 0.81; IC025 -0.22), with the outcome appearing in 7.7% of mavacamten reports versus 9.5% of comparator reports. The interval to report showed an approximately constant hazard (Weibull shape 1.00; median 161 days).

Conclusions: The crude signal does not survive restriction of the comparator to the same indication, indicating substantial confounding by indication. The restricted estimate is not evidence of safety: the comparator is small, composed largely of negative inotropes that may carry the same outcome, and subject to ascertainment and reporting biases acting in opposite directions. The magnitude of a crude disproportionality signal is therefore a poor guide to the drug's own contribution to this outcome.

## Introduction

Hypertrophic cardiomyopathy (HCM) is among the most common inherited cardiovascular diseases, with contemporary estimates placing its prevalence as high as one in 200 to one in 500 individuals [1]. The disease is characterised by pathological hypercontractility of cardiac muscle, which in a substantial subset of patients produces dynamic obstruction of the left ventricular outflow tract (LVOT) and disabling exertional symptoms.

Mavacamten (Camzyos) is a first-in-class allosteric cardiac myosin inhibitor developed to target that mechanism. In the phase 3 EXPLORER-HCM trial, it improved exercise capacity, LVOT gradient, New York Heart Association (NYHA) functional class, and symptom burden relative to placebo [2], and in VALOR-HCM, it reduced the proportion of guideline-eligible patients proceeding to septal reduction therapy [3]. The US Food and Drug Administration (FDA) approved mavacamten on April 28, 2022, for adults with symptomatic NYHA class II-III obstructive HCM. A fall in left ventricular ejection fraction was the principal safety concern in the pivotal trials, and the drug is dispensed in the United States only under a Risk Evaluation and Mitigation Strategy (REMS) that mandates baseline and serial echocardiographic assessment of ejection fraction throughout treatment [4].

Three studies have since used the FDA Adverse Event Reporting System (FAERS) to characterise the real-world safety profile of mavacamten, and each handled confounding differently. A broad survey comparing the drug against the whole database identified decreased ejection fraction as its largest disproportionality signal [5]. A second study examined atrial fibrillation and did build an HCM-specific comparator model, in which the crude signal attenuated substantially and was no longer considered significant by the authors' own Bayesian criterion [6]. A third worked within a mavacamten-treated obstructive HCM population using propensity score matching, but contrasted concomitant CYP2C19 or CYP3A4 inhibitor exposure against mavacamten monotherapy, which addresses a drug-interaction question rather than a comparison against the other drugs used for the same disease [7].

What remains untested is the comparison that bears most directly on whether mavacamten is disproportionately associated with systolic dysfunction: the drug against other agents reported to FAERS under the same HCM indication, for the systolic dysfunction and heart failure outcome cluster specifically. HCM is itself a recognised cause of systolic dysfunction and heart failure independent of any pharmacological exposure, so a whole-database comparator confounds the drug with its indication. The primary question of this study was therefore whether reports of a pre-specified systolic dysfunction and heart failure cluster are disproportionate for mavacamten relative to other agents reported under an explicit HCM indication. The whole-database comparison was retained as the crude, indication-unadjusted estimate that allows direct comparison with the earlier FAERS literature [5-7]. Two further analyses, pre-specified as exploratory descriptions rather than as tests of drug-event disproportionality, characterised the interval from treatment initiation to report and examined factors associated with a reported fatal outcome.

## Materials and methods

Study design and data source

We conducted a retrospective disproportionality analysis of individual case safety reports (ICSRs) submitted to FAERS and accessed through the openFDA application programming interface (API) [8]. The design and reporting follow the Reporting of a Disproportionality Analysis for Drug Safety Signal Detection Using Individual Case Safety Reports in Pharmacovigilance (READUS-PV) guideline [9,10]. The queries underlying the main analyses were executed without an API key between September 3 and September 8, 2026, under the public rate limits, and aggregation queries were capped at 999 results per request, a cap that does not apply to counts of unique reports, which were taken from the total returned by non-aggregated queries. All of them returned the same database snapshot date (July 30, 2026), which was verified across every response.

Exposure and outcome definitions

Mavacamten appeared in FAERS under two medicinal product names only, mavacamten (302 reports) and Camzyos (4,782 reports), with no alternative spellings detected on aggregation of co-listed product names; one report cited both. The exposed cohort was any report in which either name appeared in the drug array, yielding 5,083 unique reports. Exposure was not restricted by reported drug role, since mavacamten is rarely used as monotherapy in symptomatic obstructive HCM and is typically combined with a beta-blocker, verapamil, diltiazem, or disopyramide; restricting to reports coding it as the suspect drug would have underrepresented real-world use. Within the composite outcome, it was coded as a suspect drug in at least one entry in 355 of 379 reports (93.7%).

The composite outcome comprised 12 Medical Dictionary for Regulatory Activities (MedDRA) Preferred Terms (PTs) selected a priori: ejection fraction decreased, cardiac failure, cardiac failure congestive, left ventricular dysfunction, cardiogenic shock, ejection fraction abnormal, ventricular dysfunction, cardiac failure acute, left ventricular failure, systolic dysfunction, acute left ventricular failure, and heart failure with reduced ejection fraction. All reaction searches used the exact-match field variant (patient.reaction.reactionmeddrapt.exact), since unindexed text search on this field inflates counts for compound terms; an unindexed search for cardiac failure, for example, also matches cardiac failure congestive and cardiac failure acute. Left ventricular systolic dysfunction was evaluated as a candidate term and confirmed absent. Two further PTs, stress cardiomyopathy and cardiomyopathy, were excluded on the judgement that they most plausibly encode the underlying cardiomyopathy rather than drug-induced systolic dysfunction; both are reported descriptively in Table 1.

**Table 1:** Derivation of the study cohort from the FAERS Values are counts of unique individual case safety reports in the FAERS, openFDA snapshot of July 30, 2026. The composite outcome comprises 12 MedDRA Preferred Terms. The sensitivity-excluded terms are stress cardiomyopathy and cardiomyopathy, omitted from the composite outcome a priori. Percentages are of the exposed cohort of 5,083 reports. FDA: Food and Drug Administration; FAERS: FDA Adverse Event Reporting System; MedDRA: Medical Dictionary for Regulatory Activities

Step	Number of reports	Percentage of exposed cohort
Database and exposed cohort		
Total FAERS reports, all drugs and all events	20,692,690	Not applicable
Reports naming mavacamten or Camzyos	5,083	100.0%
Reports meeting an outcome term set		
Composite outcome, 12 Preferred Terms	379	7.5%
Sensitivity-excluded terms, 2 Preferred Terms	36	0.7%
Overlap between the two sets	9	0.2%
Union of all 14 terms	406	8.0%
Left ventricular systolic dysfunction, confirmed absent	0	0.0%

Comparators and disproportionality measures

Two comparator definitions were pre-specified. The first compared mavacamten reports against the remainder of the database; this is the crude, indication-unadjusted estimate, retained because it is the design used in the previous FAERS studies of this drug and therefore allows direct comparison with them. The second restricted the comparator to reports in which the free-text drug-indication field recorded HCM for at least one drug entry, and is the comparison that addresses the study question. Only that single unambiguous term was used; related but ambiguous terms such as cardiomyopathy, hypertensive cardiomyopathy, and cardiac hypertrophy were excluded. Because openFDA aggregates at the level of individual drug entries rather than cases, and cannot confirm that an indication entry pertains to a specific drug within a multi-drug report, the restricted analysis was built from case-level queries combining the drug search with the indication filter. Each comparator count was obtained by subtracting the mavacamten reports from all reports carrying the indication, so the comparator excludes mavacamten by construction. Per-drug estimates were not computed for the comparator, because a report may name several drugs and the indication cannot be tied to any one of them, so per-drug counts would overlap rather than partition it. Because the restricted comparator was defined by indication rather than by drug, it included reports of aficamten, a second cardiac myosin inhibitor approved by the FDA in December 2025; these reports were isolated by a targeted case-level query on the openFDA generic-name field, and a post hoc sensitivity analysis excluding them was performed in response to peer review.

Reporting odds ratios (ROR) and proportional reporting ratios (PRR) were calculated from the standard two-by-two contingency table, with 95% confidence intervals (CI) obtained on the logarithmic scale by the Woolf method and chi-square as the uncorrected Pearson statistic. Evans criteria comprise a PRR of at least 2, chi-square of at least 4, and at least three cases [11]. Because frequentist measures are unstable at low cell counts, and because the prior mavacamten literature reports Bayesian shrinkage statistics, the information component (IC) was also computed as IC = log2(O/E), where O is the observed number of reports carrying both the drug and the outcome and E = (N_drug × N_event)/N_total, with its lower 95% credibility bound obtained by the standard approximation IC025 = IC − 3.3 · O^(−1/2) − 2 · O^(−3/2). The Gamma-Poisson shrinkage estimator (empirical Bayes geometric mean, EBGM, and its lower 5% bound, EB05) requires the stratified contingency structure of the whole database and could not be fitted from aggregate API counts, so the IC is reported in its place. A signal was declared present when Evans criteria were met and IC025 exceeded zero, and absent otherwise.

Duplicate reports

FAERS contains unresolved duplicate ICSRs. Record-level deduplication would require the full quarterly data files and was outside the scope of this analysis, so the proportion of reports carrying the native duplicate flag is reported transparently rather than corrected for. Because that flag is known to miss repeat notifications, a case-matching procedure was additionally applied within the composite outcome cohort, where full case-level data were available: reports sharing an identical combination of age, sex, country, and drug start date were treated as probable repeat notifications of the same case.

Case-level review of fatal reports

As an optional READUS-PV element, reports within the composite outcome carrying the death seriousness flag were reviewed individually. The public openFDA endpoint does not include the free-text clinical narrative of an ICSR, and narrative access requires a case-by-case Freedom of Information Act request [12], so this review used the structured fields available for each report: drug role for mavacamten and all co-listed medications, indication, treatment start date, every reaction term with its individual outcome code, seriousness criteria, duplicate status, and reporter qualification.

Time-to-onset analysis

Time to onset was calculated within the composite outcome cohort as the initial FDA receipt date minus the earliest recorded start date among all mavacamten entries in that report. Reports lacking either date, or with a negative interval, were excluded; a same-day interval was recoded to 0.5 days to permit maximum-likelihood estimation. The receipt date is a proxy for, and not a measurement of, the date of event onset, because the public endpoint does not expose a reliably populated reaction-onset date. Every interval reported here therefore incorporates the unobserved delay between the clinical event and its submission, and the quantity modelled is more precisely a time to report than a time to onset. No correction was applied, since the magnitude of that delay cannot be recovered from the public data.

A two-parameter Weibull distribution was fitted by maximum likelihood with the location parameter fixed at zero, and 95% CI were obtained by percentile bootstrap with 2,000 resamples. The shape parameter was interpreted conventionally: below 1, a decreasing hazard; approximately 1, a constant hazard; and above 1, an increasing hazard. The analysis is subject to the right-truncation intrinsic to spontaneous reporting data, since reports corresponding to long true intervals may not yet have been submitted at the extraction date [13]. Rather than apply a truncation-adjusted estimator, we repeated the fit within cohorts defined by year of treatment initiation, since patients who started earlier have had more calendar time in which a long interval could be observed. A recent review argues that non-parametric distribution-comparison tests may be preferable to Weibull modelling in these data, given the absence of non-event comparators and of a defined denominator [14]; the Weibull approach was retained for comparability with the closest precedent in the mavacamten literature.

Logistic regression for fatal outcome

A logistic regression model was pre-specified to explore factors associated with a reported fatal outcome within the composite outcome cohort. Throughout, fatal outcome denotes a report carrying the FAERS death seriousness flag; it records that death was reported in association with the case and carries no causal adjudication. Because only 15 reports were so flagged, the number of covariates was constrained using the events-per-variable heuristic of approximately 10 events per covariate [15]. The model was therefore limited to age in years and sex, both chosen a priori on clinical plausibility, whilst country, indication status, and reporter qualification were examined descriptively only. Coefficients were estimated by Firth penalised regression [16] rather than ordinary maximum likelihood, to mitigate rare-event bias. Missing covariate data were handled by complete-case analysis specified a priori, with no imputation.

Statistical software

Contingency table statistics were calculated directly from openFDA query counts. The Weibull model was fitted in Python 3.11 using SciPy (Python Software Foundation, Fredericksburg, VA, USA), with bootstrap intervals computed in NumPy, and Firth regression was implemented following the modified-score algorithm [16], with Wald-type intervals from the Fisher information matrix at the penalised solution. Both models were cross-checked against a second implementation written from the underlying algorithms in a separate computing environment without SciPy or NumPy, and the two agreed to every decimal place reported here on all four published estimates.

Ethics approval

This study used only publicly available, de-identified data from FAERS, accessed through the openFDA API. Because no identifiable patient information was collected and no primary data were generated from human subjects, institutional review board approval was not required, consistent with standard practice for disproportionality analyses of public surveillance data.

Data availability

The FAERS data analysed here are publicly and freely available through the openFDA Drug Adverse Event API without access restrictions. All material required to reproduce the analysis is deposited in a public repository under DOI 10.5281/zenodo.23298751. The deposit contains every query underlying a reported figure, written out in full with its endpoint and its exposure, outcome, and indication clauses, together with the internal consistency checks performed across them; the JSON responses returned by every query of the original extraction, which fix the July 30, 2026, snapshot and allow the reported counts to be verified without reissuing the queries against a later state of the database; the case-level dataset of the 379 reports meeting the composite outcome, with its variable dictionary; and the four analysis scripts that reproduce the published estimates, together with the library-independent implementation used for cross-validation and its raw output.

## Results

Cohort derivation and characteristics

Of 20,692,690 reports in FAERS at extraction, 5,083 (0.02%) named mavacamten or Camzyos, and 379 of those (7.5%) met the composite outcome (Table 1). A further 36 reports (0.7%) matched one of the two sensitivity-excluded terms, of which nine overlapped with the composite outcome, so the union of all 14 terms comprised 406 reports (8.0% of the exposed cohort).

Among the 379 reports meeting the composite outcome (Table 2), age was recorded in 285 (75.2%), with a median of 69.0 years (interquartile range 61.0-78.0). Reports came predominantly from the United States (259/379, 68.3%) and from 20 countries in total, and physicians were the most frequent reporters (150/379, 39.6%). Most reports were serious (289/379, 76.3%), 166 (43.8%) involved hospitalisation, and 15 (4.0%) carried the death seriousness flag. Fifty-eight reports (15.3%) carried the native duplicate flag, approximately three times the proportion seen across the whole exposed cohort (271/5,083, 5.3%), consistent with more intensive follow-up reporting of the more serious cases.

**Table 2:** Demographic, reporting, and severity characteristics of the 379 reports meeting the composite outcome Values are counts of individual case safety reports unless otherwise stated, and percentages are of the cohort of 379 reports. Age percentiles are derived from the 285 reports with a recorded age. The death seriousness flag records a death reported in association with the case and carries no causal adjudication. An explicit hypertrophic cardiomyopathy indication was present in 326 reports by drug-array parsing and in 327 by case-level query, a difference of one report. FAERS: Food and Drug Administration Adverse Event Reporting System

Characteristic	Value	Percentage of cohort
Age (years)		
Reports with age recorded	285	75.2%
Reports with age missing	94	24.8%
Median	69.0	Not applicable
25th percentile	61.0	Not applicable
75th percentile	78.0	Not applicable
Minimum	21	Not applicable
Maximum	96	Not applicable
Sex		
Female	206	54.4%
Male	113	29.8%
Not reported	60	15.8%
Country of origin		
United States	259	68.3%
Countries represented	20	Not applicable
Reporter qualification		
Physician	150	39.6%
Other health professional	92	24.3%
Consumer	69	18.2%
Pharmacist	65	17.2%
Not reported	3	0.8%
Seriousness		
Serious report	289	76.3%
Hospitalisation reported	166	43.8%
Death seriousness flag	15	4.0%
Report characteristics		
Native FAERS duplicate flag	58	15.3%
Explicit hypertrophic cardiomyopathy indication	326	86.0%
Mavacamten coded as suspect drug in at least one entry	355	93.7%
Multiple mavacamten entries in the same report	135	35.6%

Disproportionality analysis

Table 3 gives the contingency counts for both comparators and Table 4 the resulting measures. Against the remainder of the database, the composite outcome was strongly disproportionate: ROR 8.29 (95% CI 7.47 to 9.21), PRR 7.75 (95% CI 7.03 to 8.54), Pearson chi-square 2,244.8, with 379 reports observed against 49.0 expected under independence. Evans criteria were met on all three components, and the Bayesian measure agreed, with an IC of 2.95 and a lower bound of 2.78. In absolute terms, the outcome appeared in 7.5% of mavacamten reports and 1.0% of all other reports.

**Table 3:** Contingency counts for the composite outcome under each comparator Values are counts of individual case safety reports. The whole-database comparator comprises all reports that do not name mavacamten. The HCM-indication comparator is restricted to reports in which the drug-indication field recorded hypertrophic cardiomyopathy for at least one drug entry and that do not name mavacamten. HCM: hypertrophic cardiomyopathy

Drug group	Reports with outcome	Reports without outcome	Total reports
Whole-database comparator			
Mavacamten	379	4,704	5,083
All other drugs	199,085	20,488,522	20,687,607
Total	199,464	20,493,226	20,692,690
HCM-indication comparator			
Mavacamten	327	3,914	4,241
All other drugs	50	477	527
Total	377	4,391	4,768

**Table 4:** Disproportionality measures for the composite outcome under each comparator Confidence intervals (CI) for the reporting odds ratio and the proportional reporting ratio are Woolf logarithmic intervals. Chi-square is the uncorrected Pearson statistic; the Yates-corrected values are 2,238.0 and 1.8, respectively. The information component is the base-2 logarithm of observed over expected counts, shown with its lower 95% credibility bound. Evans criteria require a proportional reporting ratio of at least 2, a chi-square of at least 4, and at least three cases [11]; a signal is declared when Evans criteria are met and the lower bound of the information component exceeds zero. The HCM-indication comparator arm contains 50 outcome events. In a post hoc sensitivity analysis excluding the 34 comparator reports naming aficamten, the HCM-indication comparator gave a reporting odds ratio of 0.90 (95% CI 0.64 to 1.26), a proportional reporting ratio of 0.91 (95% CI 0.67 to 1.23), a chi-square of 0.40, and a lower information component bound of -0.20. HCM: hypertrophic cardiomyopathy

Measure	Estimate	95% interval
Whole-database comparator		
Reporting odds ratio	8.29	7.47 to 9.21
Proportional reporting ratio	7.75	7.03 to 8.54
Pearson chi-square	2,244.8	Not applicable
Information component	2.95	Lower bound 2.78
Evans criteria met	Yes	Not applicable
HCM-indication comparator		
Reporting odds ratio	0.80	0.58 to 1.09
Proportional reporting ratio	0.81	0.61 to 1.08
Pearson chi-square	2.0	Not applicable
Information component	-0.04	Lower bound -0.22
Evans criteria met	No	Not applicable

Against reports sharing an explicit HCM indication, the signal was absent: ROR 0.80 (95% CI 0.58 to 1.09), PRR 0.81 (95% CI 0.61 to 1.08), chi-square 2.0, with 327 reports observed against 335.3 expected. No Evans criterion was met, and the Bayesian measure agreed, with an IC of -0.04 and a lower bound of -0.22. The outcome was recorded in 327 of 4,241 mavacamten reports (7.7%) and in 50 of 527 comparator reports (9.5%). Two features of this comparison constrain its interpretation. Mavacamten accounted for 4,241 of the 4,768 reports carrying an explicit HCM indication (88.9%), so the drug under study supplies most of the restricted database. The resulting interval remains compatible with anything from approximately 40% lower to approximately 10% higher reporting odds relative to other drugs used in HCM. Thirty-four of the 527 comparator reports named aficamten, none of them also naming mavacamten, and eight of these met the composite outcome. Excluding them, the restricted estimate was ROR 0.90 (95% CI 0.64 to 1.26) and PRR 0.91 (95% CI 0.67 to 1.23), chi-square 0.40, no Evans criterion was met, and the lower bound of the IC was -0.20.

Fatal reports

Age was available for 14 of the 15 fatal reports, with a median of 79.0 years, an interquartile range of 65.5 to 87.0 years, and a range of 35 to 96 years. Ten were female, four male, and one not reported, and mavacamten was the only drug coded as a suspect drug in all 15 (Table 5). In 10 reports, the composite-outcome term itself carried the fatal outcome code; in four, the code sat on a different but clinically related reaction term within the same report, and in one, the outcome code was missing for every term. Five of the 15 carried the native duplicate flag.

**Table 5:** Case-level review of the 15 reports within the composite outcome carrying the death seriousness flag Attribution is direct when the composite-outcome term itself carries the fatal outcome code and indirect when the code is attached to a different reaction term in the same report. Case narratives are not released through the public interface, so attribution rests on structured outcome codes and is not a causality assessment. Reports 26738146 and 26765822 share age, sex, and country and were received 10 days apart; they differ in reporter qualification, recorded indication, and number of mavacamten entries, and were not merged. EU denotes a European Union member state, an openFDA country aggregate.

Report identifier	Age (years)	Sex	Country	Duplicate flag	Fatal outcome attribution
21309625	86	F	US	Yes	Direct (acute left ventricular failure)
21707543	93	F	US	Yes	Indirect (fatal code on ventricular tachycardia)
24019909	96	Not reported	US	No	Indirect (fatal code on multiple organ dysfunction syndrome)
25213743	35	M	HU	No	Direct (acute left ventricular failure)
25407708	Not reported	F	US	No	Direct (cardiac failure)
25551874	71	M	EU	Yes	Direct (cardiogenic shock)
25667976	55	F	GB	No	Indirect (fatal code on cardiac arrest)
26051437	67	F	EU	Yes	Direct (cardiogenic shock)
26221086	90	M	US	No	Direct (heart failure with reduced ejection fraction)
26370383	76	F	EU	No	Direct (cardiogenic shock)
26571980	63	M	EU	No	Indirect (fatal code on cardiac arrest)
26697274	82	F	US	No	Direct (cardiogenic shock)
26738146	87	F	US	No	Indeterminate (outcome code missing for all terms)
26765822	87	F	US	Yes	Direct (cardiac failure congestive)
26819248	65	F	US	No	Direct (cardiogenic shock)

Time to onset

Of the 379 reports, 244 (64.4%) lacked a recorded drug start date, and one had a negative interval, leaving 134 (35.4%) for analysis. The median interval from treatment start to report receipt was 161 days, with an interquartile range of 77.5 to 420.2 days and a range of 0 to 1,206 days. The Weibull fit gave a shape parameter of 1.00 (95% bootstrap CI 0.90 to 1.13) and a scale of 272.1 days (95% CI 224.5 to 324.5), with the interval for the shape centred on unity (Table 6).

**Table 6:** Weibull model of the interval from treatment start to report receipt, with a sensitivity analysis by year of treatment initiation The modelled interval runs from the earliest recorded mavacamten start date to the initial FDA receipt date. The public interface does not expose a reaction-onset date, so the interval includes the unobserved reporting delay. The fit used 134 of 379 reports; 244 lacked a drug start date, and one had a negative interval. Confidence intervals are percentile bootstrap intervals from 2,000 resamples. Cohorts are defined by year of treatment initiation. FDA: Food and Drug Administration

Parameter	Estimate	95% confidence interval
Weibull fit, all 134 reports		
Shape	1.00	0.90 to 1.13
Scale (days)	272.1	224.5 to 324.5
Observed interval (days)		
Median	161	Not applicable
25th percentile	77.5	Not applicable
75th percentile	420.2	Not applicable
Weibull fit, treatment started 2022 or 2023, 47 reports		
Shape	0.92	Not applicable
Scale (days)	336.4	Not applicable
Weibull fit, treatment started 2024 onwards, 87 reports		
Shape	1.12	Not applicable
Scale (days)	239.1	Not applicable

Repeating the fit by year of treatment initiation showed the pattern that right-truncation predicts. Among the 47 reports with treatment starting in 2022 or 2023, the shape was 0.92 and the scale 336.4 days, whereas among the 87 starting from 2024 onwards, the shape was 1.12 and the scale 239.1 days, with the median essentially stable at 164 and 158 days, respectively. The more recent cohort yields a shorter scale because its long intervals have not yet had the opportunity to be reported, which indicates that the pooled scale of 272.1 days underestimates the true value. The shape parameter, which brackets unity in both cohorts, is the more robust quantity, and the interpretation below rests on it and on the median rather than on the scale.

Factors associated with a reported fatal outcome

Of the 379 reports, 111 (29.3%) were excluded for missing age or sex, leaving 268 reports, retaining 13 of the 15 fatal reports. Age carried an odds ratio of 1.039 per year (95% CI 0.988 to 1.092; p = 0.135) and male sex an odds ratio of 1.034 (95% CI 0.320 to 3.343; p = 0.955) (Table 7). With 13 events and two covariates, the model sits far below the conventional events-per-variable threshold even after penalisation, and the interval for sex spans a more than 10-fold range. These estimates indicate an absence of detectable association in an underpowered exploratory model rather than evidence that age and sex do not influence mortality among reported cases.

**Table 7:** Firth penalised logistic regression of reported fatal outcome on age and sex within the composite outcome cohort The model includes 268 of 379 reports and 13 of 15 fatal reports; 111 reports were excluded by complete-case analysis for missing age or sex. Fatal outcome denotes the FAERS death seriousness flag, without causal adjudication. Coefficients were estimated by Firth penalised regression to mitigate rare-event bias, with Wald-type intervals from the Fisher information matrix at the penalised solution. At 13 events, the model is exploratory and underpowered, so a non-significant estimate should not be read as evidence of no association. FAERS: Food and Drug Administration Adverse Event Reporting System

Covariate	Odds ratio	95% confidence interval	Wald p-value
Age, per year	1.039	0.988 to 1.092	0.135
Sex, male versus female	1.034	0.320 to 3.343	0.955

Sensitivity to duplicate reports

Reports sharing an identical combination of age, sex, country, and drug start date formed four clusters comprising 13 reports, of sizes 2, 3, 3, and 5, none containing a fatal report. Agreement with the native duplicate flag was inconsistent across these clusters, ranging from none of two to three of three reports flagged, which confirms that the flag alone undercounts duplication. A looser signature using age, sex, and country without a start-date match captured 196 of 379 reports (51.7%) in 58 groups; given how narrow the demographic space of this cohort is, most such matches are plausibly coincidental, and this criterion was not used for exclusion. Collapsing each strong-evidence cluster to its earliest report, leaving 370, and refitting left the Weibull and Firth estimates materially unchanged (Table 8).

**Table 8:** Weibull and Firth estimates before and after collapsing probable duplicate report clusters The sensitivity cohort excludes 9 reports forming four clusters with identical age, sex, country, and drug start date, taken as probable repeat notifications; no cluster contained a fatal report. Weibull intervals are percentile bootstrap intervals from 2,000 resamples, and Firth intervals are Wald-type intervals from the Fisher information matrix. The comparator populations of the two disproportionality analyses were not deduplicated. CI: confidence interval

Parameter	Primary estimate	Primary 95% CI	Sensitivity estimate	Sensitivity 95% CI
Weibull shape	1.00	0.90 to 1.13	0.97	0.87 to 1.09
Weibull scale (days)	272.1	224.5 to 324.5	253.6	208.0 to 308.3
Firth odds ratio, age per year	1.039	0.988 to 1.092	1.038	0.986 to 1.092
Firth odds ratio, male sex	1.034	0.320 to 3.343	0.994	0.307 to 3.216

The disproportionality estimates themselves cannot be recomputed on deduplicated comparator populations through the public interface, but the bias introduced by duplicate reports can be bounded arithmetically. Duplication shifts a ROR only when it is differential; if repeat notifications arise at the same rate in all four cells, they cancel. Within the exposed cohort, the rate is differential by outcome status, since 15.3% of reports meeting the composite outcome carry the duplicate flag against 4.5% of those that do not, or 213 of 4,704, which inflates the crude estimate. Removing duplicates from the exposed arm alone at the observed rates lowers the crude ROR from 8.29 to 7.36; if the comparator arm carries the same differential, the effect cancels and the estimate returns to 8.29, and intermediate assumptions of 5% and 10% differential duplication give 7.39 and 7.80. The crude signal is therefore bounded between approximately 7.4 and 8.3 across the plausible range, so unresolved duplication cannot account for a signal of this magnitude.

## Discussion

Mavacamten showed a strong reporting signal for systolic dysfunction and heart failure against the whole database, and no signal by either criterion once the comparator was restricted to reports sharing the same HCM indication. Frequentist and Bayesian criteria agreed in both directions, so the reversal is not an artefact of the choice of statistic. The interpretation of that reversal and its limits are the substance of this paper.

Comparison with prior FAERS studies

Situating these results against the three published analyses requires attention to which outcome and which comparator each used. Yukselen et al., analysing a snapshot covering January 2022 to September 2023 against the whole database, reported a ROR of 33.60 (95% CI 21.79 to 51.82) for decreased ejection fraction and 9.39 (95% CI 6.49 to 13.60) for cardiac failure [5]. The crude composite estimate reported here, 8.29, sits close to their cardiac failure figure and well below their decreased-ejection-fraction figure, which is the expected behaviour of a composite that pools the single highest-signal term with 11 lower-frequency terms and supports the internal consistency of the present extraction.

Ding et al. examined atrial fibrillation rather than systolic dysfunction and built both a general-population model (adjusted ROR 18.54, 95% CI 11.53 to 29.80) and an HCM-specific model in which the estimate fell to 4.28 (95% CI 1.30 to 14.07) [6]. Although that interval excluded unity, the authors did not regard it as a signal, because the lower bound of their Bayesian shrinkage estimate fell below the detection threshold, and concluded that no significant association could be identified. Their work therefore already shows, for a different outcome, the phenomenon central to this analysis: a large whole-database signal that does not survive restriction of the comparator to the shared indication. Chen et al. worked entirely within a mavacamten-treated obstructive HCM population with propensity score matching, but contrasted concomitant CYP inhibitor use against monotherapy, yielding an elevated estimate in the combination group (ROR 25.08, 95% CI 18.63 to 33.76) [7]. Their Weibull analysis of heart failure onset in that subgroup gave a shape of 0.88 and a median of 80 days, an earlier and more front-loaded pattern than the constant hazard and 161-day median seen here, consistent with pharmacokinetic potentiation acting early in treatment but drawn from a different and smaller subpopulation.

The contribution of the present study is correspondingly narrow. It is, to our knowledge, the first analysis to restrict the comparator by indication for the systolic dysfunction and heart failure outcome cluster in mavacamten, the outcome for which the drug carries a REMS, and to show that the crude signal is no longer detectable after that restriction.

Confounding by indication and the limits of the restricted estimate

HCM is not a haemodynamically neutral background against which a drug effect on systolic function can be read in isolation, since the disease itself causes systolic dysfunction and heart failure, including a well-described end-stage phenotype. In a whole-database comparison, essentially every report in the exposed cohort carries the diagnosis that predisposes to the outcome, whilst almost none of the comparator does. The crude ROR of 8.29 should therefore be read as an upper bound that is unadjusted for indication, not as an estimate of the independent contribution of the drug. For a labelled event under mandated echocardiographic surveillance and stimulated reporting, a large whole-database estimate would be expected even in the absence of any excess risk.

The restricted estimate cannot be read as evidence of safety, for three reasons. Its comparator consists almost entirely of beta-blockers, verapamil, diltiazem, and disopyramide, every one a negative inotrope capable of precipitating heart failure in this population; a comparator that plausibly carries the same outcome drives any disproportionality measure towards unity whether or not the drug under study is harmful, which is the recognised trap of active-comparator designs in pharmacovigilance. Precision is limited, since 527 comparator reports and 50 outcome events leave the interval compatible with a meaningful difference in either direction. In addition, mavacamten supplies 88.9% of the restricted database, so the drug under study constitutes most of the background against which it is judged.

Two structural biases act on this comparison in opposite directions. Camzyos is dispensed under a REMS mandating baseline and serial echocardiographic assessment of ejection fraction, required every 12 weeks during maintenance before April 2025 and every three to six months thereafter for patients on a stable dose [4]. Aficamten, the second cardiac myosin inhibitor, contributed 34 reports to the restricted comparator and is dispensed under a REMS of similar design, with echocardiography every two to eight weeks during titration and every three to six months on a maintenance dose [17]. The composite outcome appeared in eight of those 34 reports, a higher proportion than in the mavacamten arm or in the remainder of the comparator; on 34 reports accrued in the first months after launch, the proportion is too imprecise to interpret as a risk, but its direction is what protocol-mandated echocardiography and stimulated reporting of a labelled event would both predict. No comparable protocol-mandated surveillance applies to the remaining comparator agents, and that asymmetry should inflate the reporting of ejection-fraction events in the mavacamten arm, since scheduled echocardiography detects asymptomatic falls that would otherwise go unmeasured. Alone, this makes the restricted estimate conservative. Working the other way, a recently approved and commercially prominent drug attracts spontaneous reports of all kinds at rates that established generic comparators do not. That non-specific inflation enters the denominator of the exposed arm and depresses the ratio, whilst the comparator arm suffers the mirror image, since a report naming a decades-old beta-blocker is more likely to be filed when the event is unusual or severe. The restricted estimate is therefore bracketed by identifiable biases in both directions whose relative magnitudes these data cannot resolve, and is better read as evidence that the crude signal is substantially confounded than as a precise estimate of a residual drug effect.

Onset pattern

The interval from treatment start to report receipt had a median of 161 days and an approximately constant hazard, so reported events accumulated at a broadly even rate across the first years of therapy rather than clustering in the initiation and titration window. Two readings are possible and cannot be separated here. A biological reading would place the risk of ejection-fraction decline beyond dose escalation and into maintenance therapy, which argues for sustaining rather than relaxing long-term surveillance. A procedural reading notes that echocardiography was mandated every 12 weeks during maintenance for most of the study period, and that a detection process running on a fixed calendar will itself generate an approximately uniform distribution of detection times whatever the underlying hazard. The 2025 extension of the maintenance interval to every three to six months [4] offers a natural experiment that future extractions could exploit: if the onset distribution is largely an artefact of the monitoring calendar, its shape should change measurably in reports accruing after that label change, whereas a biologically driven hazard should be unaffected.

Strengths and limitations

This study has several strengths. The outcome was defined a priori with exact-match terms, which avoids the inflation of compound PTs that unindexed search produces. Frequentist and Bayesian measures were computed and agreed in both comparisons. The bias introduced by duplicate reports was bounded arithmetically rather than set aside, and the Weibull and Firth estimates were reproduced by a second implementation built from the algorithms rather than from a shared library.

This study shares the limitations inherent to any disproportionality analysis of spontaneous reporting data. The absence of an exposure denominator precludes calculation of incidence or true relative risk; reporting is voluntary and subject to stimulated reporting and underreporting, and causality cannot be established from an aggregate measure. Several limitations are specific to this analysis. The free-text indication field used to define the restricted comparator is neither standardised nor complete, and openFDA cannot confirm that a given indication entry pertains to a specific drug within a multi-drug report, so residual confounding by indication cannot be excluded; the one-report discrepancy between retrieval paths noted under Table 2 illustrates that imprecision concretely. The restriction applied here is by indication string, not by treatment: the comparator comprises reports in which HCM appears on at least one drug entry, without confirmation that the indication attaches to the comparator drug, and without separation of obstructive from non-obstructive disease or any measure of severity. It therefore does not balance channelling by phenotype or disease stage. The composite outcome is also coarser than the interpretation requires. Decreased ejection fraction is the term under protocol-mandated surveillance, and scheduled echocardiography should enrich it specifically, whereas cardiogenic shock and the overt heart failure terms are not detected that way; no per-term estimates are reported for the indication-restricted comparison, so a single null composite cannot show that these behave alike. No suspect-only or monotherapy sensitivity analysis was performed either, although 35.6% of outcome reports carried more than one mavacamten entry and co-medication with agents that can themselves worsen systolic function was routine. Apart from aficamten, whose reports were excluded in a sensitivity analysis, per-drug estimates were not computed for the comparator, so it is not possible to determine whether another agent drives its outcome proportion, and the Gamma-Poisson shrinkage estimator used in two of the three prior studies could not be fitted from aggregate counts.

The time-to-onset analysis rests on receipt date as a proxy for event onset, is subject to right-truncation, and used only the 35.4% of reports carrying a drug start date, a subset that may differ systematically from the remainder. The regression was limited in advance to two covariates given 15 fatal reports, retains 13 after complete-case exclusion, and is exploratory; its null estimates reflect low power rather than demonstrated absence of association, and the fatal outcome flag records a reported death without causal adjudication. The duplicate-clustering check applies only to the case-level cohort, so the disproportionality estimates remain uncorrected for duplication and the reported duplicate proportions are a lower bound. Finally, cross-validation against the R reference implementations was not possible in either computing environment available; the agreement of two independent implementations, together with the deposited code, was judged sufficient for an exploratory analysis, but is disclosed as a methodological choice rather than an ideal.

## Conclusions

Mavacamten showed a strong crude disproportionality signal for systolic dysfunction and heart failure in FAERS. That signal was no longer detectable once the comparator was restricted to reports sharing the same HCM indication, which indicates that confounding by indication accounts for a substantial part of the crude estimate. The restricted estimate should not itself be read as evidence of safety, given a small comparator composed of agents that may carry the same outcome and identifiable ascertainment and reporting biases acting in opposing directions. The magnitude of a crude disproportionality signal is therefore a poor guide to the underlying association in this setting, and the present findings are hypothesis-generating rather than confirmatory. The composite null does not separate decreased ejection fraction, the term under protocol-mandated surveillance, from overt heart failure, and channelling by disease severity and obstructive phenotype is uncontrolled. Nothing in this analysis bears on the case for the protocol-mandated echocardiographic surveillance that defines safe clinical use of mavacamten.
